# Requirements of health professionals and affected persons for an App-based dual-task training for hearing impaired older adults - a Delphi survey

**DOI:** 10.1186/s11556-025-00386-7

**Published:** 2025-10-23

**Authors:** Bettina Wollesen, Meghan Ambrens, Anna Wunderlich, Kim Delbaere

**Affiliations:** 1 German Sport University Cologne, Cologne, Germany; 2 Technische Universität Berlin, Berlin, Germany; 3 Neuroscience Research Australia, Sydney, Australia; 4 UNSW Sydney, Sydney, Australia

**Keywords:** Hearing impairments, Older adults, Digital intervention, Cognitive-motor training, Delphi study, Exercise preferences, User-centered design

## Abstract

**Background:**

Age-related hearing impairments significantly impact social interactions and physical activities for older adults. Recent studies have highlighted potential benefits of cognitive-motor training but lack specificity regarding the unique needs of this population. This study aimed to determine the preferences and specific needs of older adults with hearing impairments to develop tailored cognitive-motor training systems.

**Methods:**

A three-round Delphi survey was conducted involving health and exercise professionals (round 1: *n* = 18; round 2: *n* = 12; round 3: *n* = 11) and older adults with hearing impairments (round 1: *n* = 18; round 2: *n* = 14; round 3: *n* = 15). Round 1 collected qualitative input; rounds 2 and 3 assessed relevance and agreement of the items (≥ 70% agreement) using structured questions. Descriptive Statistics analyses and one-way ANOVA were used to compare responses between expert and user responses.

**Results:**

The study gathered 570 responses that were clustered in 148 items and 35 were removed due to low relevance. Key themes included app features, exercise characteristics, especially dual-task combinations, feedback and monitoring, as well as barriers and facilitators. While revealing a high level of consensus the data showed significant differences between the groups. For example, in the preference for training types, health professionals differed from hearing-impaired participants. While professionals emphasised the integration of sensory challenges and dual-task exercises, participants preferred less demanding physical activities and those incorporating music or ambient sounds. Strength exercises and prescribed frequent sessions were deemed less feasible, indicating a preference for more adaptable and enjoyable training regimes.

**Conclusions:**

Digital dual-task training programs for hearing-impaired older adults should be tailored to individual capabilities and preferences. Programs that are flexible, easy to use and support autonomy may promote greater engagement. Integrating sensory accommodation and user-centred design could enhance physical and cognitive outcomes, contributing to improved independence and quality of life.

**Supplementary Information:**

The online version contains supplementary material available at 10.1186/s11556-025-00386-7.

## Introduction

Age-related hearing impairment affects approximately 50% of adults over the age of 65 [1] and is expected to affect 8% of the global population by 2050 [2]. Hearing impairment adversely impacts social and emotional well-being [3] and restricts daily activities [4–7], thereby increasing the public health burden worldwide. Hearing impairment has been associated with declines in both cognitive [8–10] and physical function [6, 11], affecting gait and balance. The impacts of hearing impairment are particularly evident during dual-task activities, such as walking while performing a cognitive task [12, 13]. These situations increase the cognitive load and are associated with a higher risk of falls [14, 15], a leading cause of injury and loss of independence among older adults with hearing impairments [16].

Cognitive-motor interventions - often referred to as dual-task training - have been recommended to mitigate these risks [17]. A systematic review and meta-analysis suggested that cognitive-motor interventions can strengthen executive function, including inhibitory control, working memory, and cognitive flexibility, as well as improve static and dynamic balance in older adults [17, 18]. These benefits are particularly relevant for those with age-related hearing impairment, as dual-task training addresses unique sensory and functional challenges by enhancing sensory integration alongside balance and cognitive function [19, 20]. Despite these benefits, existing research on dual-task training tailored for older adults with hearing impairments is limited. This population requires targeted strategies, such as auditory-based exercises that involve responding to tones while maintaining balance, rather than traditional balance training approaches. Furthermore, to date there is no consensus regarding the optimal design, intensity and progression of such interventions, as well as a lack of diagnostic tools needed to assess their efficacy.

Amidst the growing relevance of digital interventions in health promotion, mHealth programs provide an excellent opportunity for delivering such tailored interventions without the constraints of locations or time [21], reaching individuals who may not have access to gyms or physiotherapy services. Furthermore, the integration of motivating features such as progress tracking and gamification within mHealth applications have potential to maintain engagement and encourage long-term use. However, the effectiveness of these interventions relies on a user-centered design (UCD), which requires consultation with users throughout the development process to ensure accessibility, usability, and motivation, especially people with cognitive or motor impairments. Therefore, it is important not only to integrate experts’ recommendations for relevant exercises and their characteristics but also compare whether these recommendations meet the needs of older adults with hearing impairments.

In summary, less is known about the specific requirements of older adults with hearing impairments and hearing related balance decrements to design a tailored mHealth intervention and related diagnostic system. This lack of knowledge comprises relevant training characteristics, potential App or system features (e.g. for feedback or monitoring of the training) as well as potential barriers regarding technical issues.

Therefore, this study aims to address these gaps through engaging both health professionals and participants with hearing or balance impairments and get knowledge about the preferences and specific needs of older adults with hearing impairments to develop tailored cognitive-motor training systems.

The main research aims are to identify and prioritise key training components, diagnostic tools and delivery methods within a three round Delphi survey.

The main research questions are:

- Which motor and hearing exercise key components should be combined for a dual-task training app?
- How should these exercises be presented and monitored?
- Which requirements and barriers have to be considered within such a digital training system?

And as a secondary aim:

- Do the preferences for the training system differ between the hearing impaired participants and health professionals?

Based on this process we establish a framework for evidence-based, user-centered mHealth training programs that enhance mobility, balance and cognitive function in older adults with hearing impairments.

## Methods

### Study design

Within the field of health science, Delphi processes serve as a systematic and methodologically rigorous approach to achieving expert consensus on a specific issue or research question. This technique is particularly valuable for synthesizing the collective knowledge and judgments of professionals as well as patients, thereby facilitating evidence-informed decision-making and addressing complex challenges that necessitate agreement across disciplinary boundaries. Its utility is especially evident in contexts where direct interaction among participants is impractical [22].

This study integrated insights from health professional experts and older adults with hearing impairment. The first survey round involved separate surveys for each participant group to address specific perspectives and complexities. Subsequent rounds integrated responses from both participant groups to refine and validate the findings. The respective surveys were provided in German or English. Each survey round adhered to ethical standards outlined in the Declaration of Helsinki [23]. Data collection was conducted online between December 2023 and July 2024.

This work was part of the “GehHörBalance” project and contributed to the development of an innovative dual-task training system that integrates auditory and motor exercises tailored to older adults with hearing impairment.

## Participants

### Sample size

We invited 220 international health and exercise science professionals as health professional experts and 30 older adults with hearing impairment. These group sizes were based on our experience on Delphi processes. Previous studies had a response rate of 15–20% among health professional experts, while for the target group higher response rates were expected [24, 25].

Following published recommendations we aimed for 8 to 15 participants for each group to meet validity thresholds [22; 26].

### Inclusion criteria

Participants included professionals with expertise in medical and therapeutic support relevant to older adults with hearing impairment, exercise delivery in older adults with or without hearing impairment, and digital solutions for older adults. Older adults were required to be at least 50 years old, and either experiencing mild to moderately severe hearing loss or associated balance issues.

### Identification of participants

The participants were identified through the broader network of the authors. Experts were invited via email based on their authorship of published research articles in this area of interest. Older adults were predominantly participants of former studies run in the same lab, who gave written consent to be contacted again in case of future studies. We also asked all recruited participants to indicate hearing impaired people they believed were suitable and who could be approached for participation in the study. At the start of each survey in every round, we asked all participants for their informed consent, so also later joining participants were covered.

### Study flow

The initial recruitment and ongoing engagement were managed through personalised email communication and reminders to encourage complete participation across all survey rounds. Participant contributions were acknowledged in the study outputs, with all participants given the option to be listed in the acknowledgments section. This inclusive approach aimed to enhance the validity and applicability of the research findings to real-world settings.

### Survey process

The surveys were administered using LimeSurvey software (LimeSurvey GmbH, Hamburg, Germany), ensuring data anonymity and security. The design and content of the surveys were pre-tested by volunteers, including hearing-impaired older adults, to ensure clarity and accessibility. The survey for the national and international experts was provided in English while the survey for the persons with hearing and/or balance impairment living in Germany was in German. The process involved initial email contact for recruitment, followed by electronic informed consent in a separate survey that, in a nested way only after agreement, led to an independent survey with the respective questions. This way, it was possible to keep all personal information (names, email addresses, consent) totally separate from the responses. The Delphi survey consisted of three rounds. There were open-ended questions in the first and second round and closed-ended questions in the second and third round following the guidelines provided by Trevelyan and Robinson [22]. Each survey phase was open for 2–3 weeks, with intervals of 2–4 weeks between rounds for data analysis and preparation of subsequent surveys.

Round 1 focused on gathering a broad range of opinions through open-ended questions covering training intervention characteristics, App functionality and barriers to implementation. Participants responded to questions across four blocks: training characteristics (*n* = 7), program functionality (*n* = 5), barriers and safety aspects (*n* = 4) and additional comments and demographics (*n* = 3). Hearing-impaired participants were specifically asked to outline their requirements and wishes, where applicable, ensuring tailored feedback directly from the target user group (cf. Table 2). More information is available in the supplemental materials.

Round 2 followed a mixed-methods format [22, 26]. Participants rated the relevance of each of the synthesised items from the first round on a 10-point Likert scale (10 points equals highest relevance). A following free-text response option allowed for participants to provide additional qualitative feedback, elaborate on their ratings, or highlight missing aspects. All items that were rated by at least 70% of the participants with a rating higher than 7.0 were summarised for final consensus in the third round. This threshold was set according to prior Delphi process experience of the author team and in line with recommendations by Diamond et al. [27].

Round 3 used closed-ended questions and aimed to reach a consensus by all participants, enabling us to define final recommendations for the App’s development. Participants were asked to specifically assess the features critical for the target user group of older hearing and/or balance impaired adults. Participants rated their agreement to all relevant items from round two on a 10-point scale from ‘totally disagree’ to ‘totally agree’. This group agreement represents the consensus on the recommended features and training elements for the App. To highlight the importance of direct user input in the development of health-related technologies, the ratings provided by hearing-impaired participants were given particular emphasis. Items with an average rating of M = 7.0 or higher from this group were prioritised and listed in the final consensus tables.

### Data analysis

Qualitative data from the first and second rounds were analysed using thematic analysis to categorise responses following the inductive approach recommended by Mayring [28]. Quantitative analysis of the ratings in Rounds 2 and 3 involved calculating frequencies, means and standard deviations. Statistical differences between the responses of health professionals and hearing-impaired participants in round 3 were analysed by using one-way ANOVAs in SPSS (IBM SPSS Statistics, Armonk, NY, USA). The significance threshold was defined at a two-tailed alpha level of 0.05. The assumption of normality was evaluated using the Kolmogorov–Smirnov test. Effect sizes are reported as partial eta squared (ηp²), with thresholds for interpretation as follows: small (ηp² ≥ 0.08), medium (ηp² ≥ 0.20), and large (ηp² ≥ 0.32) [29].

## Results

### Sample characteristics

**Fig. 1:**
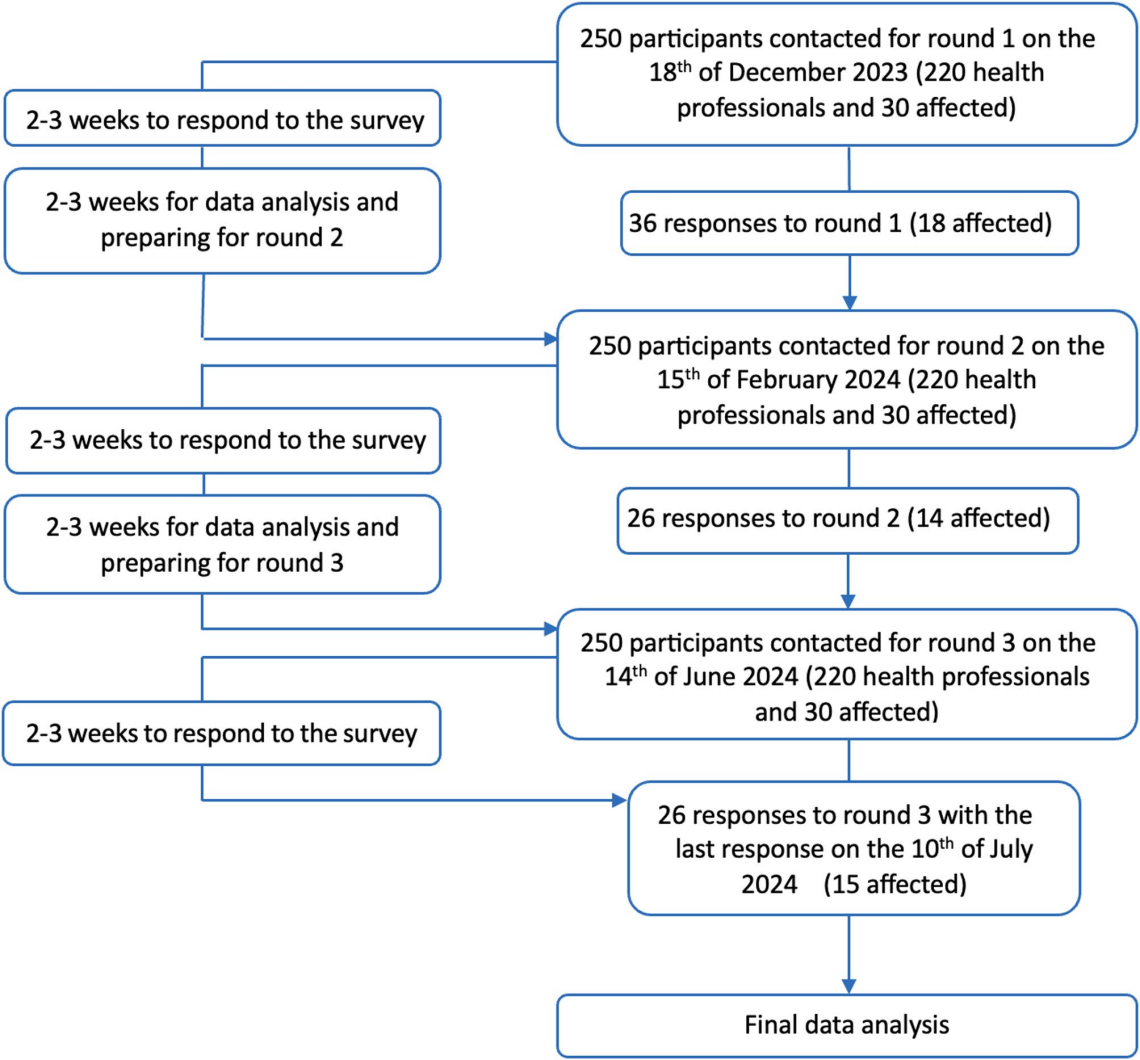
Study flow and participation

The response rate for the first round was 14% (8.3% for the experts and 60% for the older adults), and the rate of complete answers from total participation was 75%. The distribution of incomplete and complete answers is displayed next to the total participation per round and the distribution of professions among the participants is presented in Table 1. Out of a total of 250 invited persons, 36 confirmed the privacy policy in the first round. In the first round, the experts were a group of 10 females, 2 males, and 1 person who did not prefer to state their gender with an average age of 48.1 years ranging from 35 to 70 years. The group of older adults consisted of 8 females, 1 diverse person, and 4 males with an average age of 67.1 years ranging from 55 to 87 years. All responses from all three stages were included in the analysis (Fig.1).

**Table 1:** Overview of the participants of the Delphi surveys round 1–3 divided by group

	Participation rates hearing impaired older adults			Impairment			
Round	Incomplete	Complete	Total	Hearing impaired	Balance impaired	Both	Not reported
1	4	14	18	9	1	2	6
2	2	12	14	8	0	4	2
3	3	12	15	7	0	2	6

	Participation rates health professional experts			Professions			
Round	Incomplete	Complete	Total	Scientist	Physiotherapist	Physician	Not reported
1	5	13	18	6	4	1	7
2	2	10	12	5	3	2	2
3	1	10	11	6	3	1	1

### Round 1 qualitative analysis

The qualitative analysis from the first round led to 148 different aspects regarding the training characteristics as well as the App’s design and functionality, as detailed in Table 2. Health professionals provided more feedback on training content, while hearing-impaired adults offered more insights on training characteristics and specific requirements for the App. Questions related to App features and functions elicited more qualitative responses from health professionals, whereas hearing-impaired participants commented more on issues about monitoring motor and hearing functions.

**Table 2:** Overview of qualitative items related to the initial questions

No	Question	Number of responses health professionals *n* (%)	Number of responses hearing affected *n* (%)	Total
1	What features of physical training or exercise would you recommend for the target group of older hearing-impaired and/or balance-impaired adults? Please explain your answer.	43 (66.2)	22 (33.8)	65
2	What hearing exercise would you recommend for this target group of older hearing-impaired and/or balance-impaired adults? Please explain your answer.	11 (73.3)	4 (26.7)	15
3	What exercises are clinically relevant for this target group? Please explain your answer.	43 (100)	//(0)	43
4	What recommendations would you give to combine physical and hearing exercises, e.g., as a Dual-task training? Please explain your answer.	36 (90)	4 (10)	40
5	Please outline the training type, intensity, frequency and training duration you recommend for this target group. Please explain your answer.	10 (30.3)	23 (69.7)	33
6	Please outline the training type, intensity, frequency and training duration you recommend for this target group. Please explain your answer.	12 (50)	12 (50)	24
7	Please outline any training control parameters you would integrate into your treatment for this target group. Please explain your answer.	15 (100)	//(0)	15
8	How should the instructions for the training tasks be presented to the target group? What feedback would you integrate into the App? Please explain your answer.	20 (40.8)	29 (59.2)	49
9	How should the instructions for the training tasks be presented to the target group? What feedback content would you integrate into the App? Please explain your answer.	8 (40)	20 (60)	28
10	How should the instructions for the training tasks be presented to the target group? How would you present feedback in the App? Please explain your answer.	//(0)	23 (100)	23
11	What other function(s), e.g., gamification or reminders would you like to see included in an App? Please explain your answer.	31 (75.6)	10 (24.4)	41
12	What specific requirements do we have to consider if we design an App or digital intervention to improve motor functioning and hearing for older people with hearing and/or balance impairments? Please explain your answer.	26 (72.2)	10 (27.8)	36
13	What specific tests to measure motor function would you recommend integrating into the App? Please explain your answer.	14 (40)	21 (60)	35
14	What specific tests to measure hearing performance would you recommend integrating into the App? Please explain your answer.	3 (11.5)	23 (88.5)	26
15	Can you identify any barriers preventing people from following the recommendations provided in a training App that combines auditory training with movement exercises? Please explain your answer.	30 (85.7)	5 (14.3)	35
16	What concerns do you have regarding the safety and privacy of the target group when using a training and therapy App? If so, do you have ideas on how those concerns can be addressed? Please consider the target group in your answer.	21 (63.6)	12 (36.4)	33
17	What kind of data would you want to see in the back end of the App? How should this data be presented, interpreted, displayed? Please explain your answer.	29 (100)	//(0)	29
Sum		352 (61.8)	218 (38.2)	570

### Round 2 relevance of the identified items

After clustering the answers of Round 1, all 148 items were reviewed by both groups in Round 2. Participants rated the relevance of these items using a 10-point Likert scale (10 points equals highest relevance), and the 113 items that were rated by 70% of all responders with at least a rating of 7.0 are presented in Table 3. Only 35 items were removed based on the threshold after Round 2 showing a high consensus among all responders.

**Table 3:** Items per category transferred from round 2 into round 3

Category	Round 2	Round 3
Physical training or exercises	12 items	11 items
Hearing training or exercises	12 items	9 items
Dual-task training	12 items	7 items
Training frequency	8 items	3 items
Training duration	5 items	1 items
Training control measures	8 items	7 items
Instruction presentation	6 items	4 items
Feedback content	7 items	6 items
Feedback presentation	6 items	5 items
App functions	17 items	11 items
App usability	15 items	15 items
Motor function to monitor	6 items	6 items
Hearing function to monitor	9 items	9 items
Barriers of an App	16 items	10 items
Safety aspects	4 items	4 items
Interface to professionals	5 items	5 items
Summary of Items	148	113

### Round 3 consensus on the final app contents

A total of 113 items were included in Round 3 for final consensus voting. Tables 4, 5, 6 and 7 present the final results of the consensus from both groups, organised across four categories: (1) training content (consensus for *n* = 34 items; cf. Table 4), (2) feedback and monitoring (consensus for *n* = 28 items; cf. Table 5), (3) App features and barriers (consensus for *n* = 31 items; cf. Table 6), and (4) data protection and safety (consensus for *n* = 8 items; cf. Table 7). Only seven items did not reach the consensus level of 7.0.

**Table 4:** Agreement on the potential training contents of the DT training app

Question Item	Rating affected persons Mean + SD	Rating Health professionals Mean + SD
***Content to improve motor performance***		
1. Flexibility exercises (e.g., gymnastics; specific exercises for foot flexibility)	8.3 ± 1.45	7.3 ± 1.6
2. Movement combined with visual tasks (e.g., walking and observing something)	8.2 ± 1.8	8.7 ± 2
3. Movement combined with coordination tasks (e.g., combining walking with arm movements)	8.0 ± 1.9	8.5 ± 2
4. Combined training exercises with strength, endurance, flexibility, and balance	7.9 ± 2	8.5 ± 1
5. Endurance exercises (e.g., walking)	7.7 ± 2.3	8.0 ± 2.2
6. Combined strength and balance exercises for fall prevention*	7.8 ± 1.9	9.3 ± 1
7. Balance exercises (e.g., longer one-legged stance or shifting positions from right to left leg)*	7.8 ± 2	9.5 ± 0.7
8. Everyday training tasks (e.g., getting up from a chair), functional training	7.5 ± 2.7	9.1 ± 2.1
9. Exergames (computer-based games that promote movement, especially balance and strength)	7.5 ± 2.1	7.7 ± 2.6
10. Exercises with closed eyes	7.5 ± 2	8.3 ± 1.2
***Content to improve hearing performance***		
11. Exercises for distinguishing multiple voices	7.8 ± 2.1	8.8 ± 1
12. Listening exercises with background noise*	7.7 ± 2.2	10
13. Everyday exercises (e.g., understanding content from a phone call)	7.7 ± 2	9.7 ± 1
14. Combination of multiple concurrent listening tasks (e.g., tracking different sound sequences)	7.7 ± 2	9.2 ± 1
15. Exercises that involve both brain hemispheres (e.g., recognizing direction of a sound)	7.4 ± 2.8	9.8 ± 0.5
16. Exercises with and without hearing aids*	7.2 ± 2.7	9.5 ± 1
17. Exercises for recognizing sound patterns or noises	7.2 ± 2	9 ± 1
18. Exercises for recognizing consonants	7.1 ± 2.3	8.7 ± 1
19. Exercises for distinguishing pitch*	7 ± 2	9.3 ± 1
***Content for DT exercises***		
20. Navigating complex environments (e.g., crossing a street)	8.6 ± 2	9.1 ± 2.1
21. Performing exercises with music or background noise	8.4 ± 2	8.1 ± 2
22. Exercises in combination with self-efficacy training (gradually building confidence in one’s abilities)	7.6 ± 2.7	8.7 ± 2.1
23. Having conversations while walking	7.6 ± 2.2	8.9 ± 1
24. Listening tasks during balance exercises (static, e.g., standing on one leg for an extended period, and dynamic, e.g., switching from right to left leg)*	7.3 ± 2.6	9.1 ± 1
25. Exergames: computer-based games that promote movement (e.g., combining balance and hearing abilities)	7.3 ± 2.6	8.1 ± 2.2
26. Everyday exercises (e.g., walking and using the phone)*	7 ± 2.6	9.6 ± 1
***Content for training design (training characteristics; principles)***		
27. Adjustment of training intensity (e.g., complexity of tasks) based on individual physical performance and hearing ability	8.6 ± 1.8	9.4 ± 0.8
28. Increase in training difficulty based on individual improvements	8.2 ± 2	9.1 ± 1.4
29. Adjustment of training volume (e.g., duration of training sessions) based on recommendations for fall prevention	8.3 ± 1.6	8.7 ± 2.4
30. Adjustment of training volume (e.g., duration of training sessions) based on individual physical and cognitive performance	8.0 ± 2.4	8.8 ± 2.5
31. Recording of training participation	7.8 ± 2.4	8.7 ± 1
32. 4–5 times per week	7.7 ± 2.6	8.4 ± 2.8
33. Regular tests to assess individual physical fitness and hearing ability	7.5 ± 2	7.3 ± 2.5
34. Training cycle of at least 20 weeks	7.3 ± 2.1	7.5 ± 2

Legend: Items with an * showed significant differences between the groups

**Table 5:** Agreement on training instruction, feedback and monitoring

Question Item	Rating Hearing affected person Mean + SD	Rating Health professionals Mean + SD
***Instruction***		
1. Combination of short videos and explanatory text	8.5 ± 2	9.6 ± 0.7
2. Combination of images and text	8 ± 2.2	8.7 ± 2.5
3. Short videos	7.7 ± 2.6	8.6 ± 0.8
4. Images	7.2 ± 2	7.6 ± 2.2
***Feedback***		
5. Available at any time	8.4 ± 1.8	7.9 ± 2.7
6. Feedback on current performance compared to the goal	8.1 ± 1.9	8.6 ± 2.1
7. Presentation format: Visual (e.g., pop up of points gained)	8 ± 3	7.6 ± 2.5
8. Feedback on current performance	7.8 ± 1.8	8.8 ± 0.7
9. Correction during the execution of exercises	7.6 ± 2.7	8.9 ± 1
10. Feedback on current exercise progress (e.g., number of exercises completed)	7.5 ± 2	8.4 ± 1.5
11. Feedback Timing: Weekly	7.3 ± 2.9	7.6 ± 2.5
12. Feedback after each session	7.1 ± 3	7.6 ± 3
13. Feedback on the difficulty level of the exercise	7.1 ± 1.9	8 ± 2
***Monitoring***		
14. Hearing everyday sounds despite various background noises	8.9 ± 1.7	9.5 ± 1
15. Hearing everyday sounds, hearing threshold (e.g., PTA)	8.8 ± 1.7	9.5 ± 1
16. Speech discrimination	8.7 ± 1.8	9.8 ± 0.5
17. Word recognition in background noise	8.7 ± 1.7	10
18. Risk of falls	8.6 ± 2	9.4 ± 0.8
19. Number of steps per day	8.1 ± 2.8	8.1 ± 1.1
20. Sound localization	8.2 ± 1.7	9.8 ± 0.0
21. Recognition of the content of spoken words	8.1 ± 3.1	10
22. Leg mobility	8 ± 2.1	7.3 ± 1.7
23. Postural stability/balance	8 ± 2.6	9.2 ± 0.8
24. Reaction speed to signals	7.8 ± 2.6	8.6 ± 0.9
25. Combination of strength, balance, and gait (e.g., SPPB or TUG)	7.7 ± 2.8	9.3 ± 0.7
26. Sensitivity to tone discrimination	7.7 ± 3	9.3 ± 1
27. Auditory memory	7.6 ± 2.4	9.3 ± 1
28. Gait analysis (e.g., speed, posture, safety)	7.4 ± 2.7	8.2 ± 0.8

**Table 6:** App requirements, user barriers and features

Question Item	Rating Hearing affected person Mean + SD	Rating Health professionals Mean + SD
***Requirements of the App design and its functionalities***		
1. Options for integrating personal hearing aids	9.1 ± 1.7	8.7 ± 1.9
2. Ease of use	9.1 ± 1.7	9.3 ± 1.4
3. User manual	9 ± 1.7	7.1 ± 2.5
4. Compatibility with existing devices	9 ± 1.7	9 ± 1.4
5. Easily understandable instructions	8.9 ± 1.6	10
6. Support for learning how to use the App upon initial use	8.8 ± 1.7	9.2 ± 1
7. Exercises that are enjoyable	8.7 ± 1.7	8.9 ± 1.5
8. Option to receive technical support	8.7 ± 1.7	9.1 ± 1.5
9. Simple graphics for monitoring progress	8.4 ± 1.6	9.3 ± 1
10. Simple tests to assess hearing ability	8.4 ± 1.5	8.8 ± 1.2
11. Instructions for creating a safe training environment	8.3 ± 2	9.4 ± 0.7
12. Simple tests to assess physical fitness	8.1 ± 1.9	9.1 ± 0.8
13. Simple barrier-free design (e.g., large letters and input buttons)	8 ± 2	9.4 ± 0.7
14. Movement tracking	8 ± 1.8	7.7 ± 1
15. Option for training without additional functions	7.4 ± 2.8	9.1 ± 1.2
***Barriers for users***		
16. Low self-efficacy	8.3 ± 2.2	8.4 ± 1.5
17. Poor user-friendliness*	7.6 ± 2.4	9.8 ± 0.7
18. Lack of clear or absent rationale for recommendations	8 ± 2	8 ± 2
19. Feeling overwhelmed/Frustration	7.8 ± 2.1	9.2 ± 1.3
20. Too high complexity of the App and its operation*	7.3 ± 2.8	9.8 ± 0.7
21. Time and opportunity	7 ± 2.9	7.9 ± 2.2
***Functionalities and features***		
22. Opportunities for networking with medical care (e.g., personal counseling)	8.3 ± 2	8.8 ± 1
23. Buttons for repeating instructions	8.3 ± 2.4	9.6 ± 0.8
24. Option to select the format (verbal, video, or written) for exercise instructions*	7.9 ± 2.3	9.8 ± 0.7
25. Reminder function	7.8 ± 1.9	8.3 ± 2.3
26. Display of current heart rate and calorie expenditure	7.9 ± 3.1	6.1 ± 2.8
27. Information on techniques for behavior change	7.4 ± 2.4	7 ± 2
28. Information material on hearing impairment and balance difficulties	7.6 ± 2.3	6.8 ± 2
29. Information on how training adjustments are made	7.6 ± 1.7	8.4 ± 2
30. Specific elements to enhance motivation (e.g., positive messages)	7.4 ± 2.8	8.8 ± 1.3
31. Option to select the format (verbal, video, or written) for exercise feedback	7.3 ± 3.2	9.3 ± 1.3

Legend: Items with an * showed significant differences between the groups

**Table 7:** Data protection and safety

Question Item	Rating Hearing affected person Mean + SD	Rating Health professionals Mean + SD
1. Safety instructions for use	8.6 ± 1.6	8.9 ± 1.3
2. Safety instructions during training, warnings	8.1 ± 2	8.9 ± 1
**Voluntary submission of data to professionals**		
3. Physical fitness	8.2 ± 2	8.6 ± 1
4. Training results/success	8.1 ± 2	8.7 ± 2
5. Training participation/duration	8 ± 2	9.4 ± 1
6. Thresholds for clinically relevant changes	7.7 ± 3	9.3 ± 1
7. User satisfaction feedback	7 ± 2	8.1 ± 2

### Training contents

 The analysis revealed notable differences in how health professional experts and hearing-impaired older adults rated training components. Health professionals typically rated motor-related items, such as “Combined strength and balance exercises for fall prevention” (F(1,22) = 5.307; *p* = 0.031; _p_eta^2^=0.194) and “Balance Exercises” (F(1,22) = 8.208; *p* = 0.009; _p_eta^2^=0.272), significantly higher than hearing-impaired participants (cf. Table 4). Similar trends were observed for hearing-related items “Listening exercises with background noise” (F(1,17) = 6.243; *p* = 0.025; _p_eta^2^=0.294), “Exercises with and without hearing aids” (F(1,17) = 5.676; *p* = 0.031; _p_eta^2^=0.275) and “Exercises for distinguishing pitch” (F(1,16) = 6.427; *p* = 0.023; _p_eta^2^=0.300). The dual-task (DT) training items, such as “Listening tasks during balance exercises (static, e.g., standing on one leg for an extended period, and dynamic, e.g., switching from right to left leg)” (F(1,20) = 4.675; *p* = 0.044; _p_eta^2^=0.197) and “Everyday exercises” (e.g., walking and using the phone) (F(1,20) = 9.163; *p* = 0.007; _p_eta^2^=0.337), ratings from health professionals were also higher. Items that did not meet the threshold of a 7.0 rating, such as “Strength exercises (e.g., squats)” were excluded from the final recommendations.

The items “Presentation of performance curves” and the item “Feedback after each exercise” were excluded as they did not meet the thresholds of > 7.

### App features and barriers

The following items, identified under barriers to using a training App, had significant differences: “Poor user-friendliness” (F(1,20) = 7.143; *p* = 0.017; _p_eta^2^=0.309) and “Too high complexity of the App and its operation” (F(1,20) = 6.368; *p* = 0.022; _p_eta^2^=0.285) and showed lower ratings by hearing-impaired adults (cf. Table 6). The feature “Option to select the format (verbal, video, or written) for exercise instructions” differed between the two groups (F(1,20) = 5.985; *p* = 0.027; _p_eta^2^=0.176), with higher ratings from the health professionals. “Too high complexity of the App and its operation”, along with “incompatibility with older smartphone models”, “Lack of technology/equipment”, “Low willingness to use smartphones” and “Visual impairment” were excluded due to ratings < 7 by the hearing-impaired adults. Also the items “Gamification elements (e.g., high scores, rewards for participation and achievements, etc.)” and “Competitions with other users” were excluded due to scores < 7 by the hearing-impaired older adults.

*Data protection and safety*. All data protection items were rated higher by the health professionals. However, the item “Legal data protection including information about the collection and use of personal data” was removed due to an overall rating < 7, reflecting possible concerns or differing priorities between the groups regarding data security measures (cf. Table 7).

## Discussion

This Delphi study explored the design requirements for an mHealth App system delivering cognitive-motor training for older adults with hearing impairments. By involving both health professionals and the target demographic, this research sought to create a robust, evidence-based framework that addresses the unique challenges this group faces in maintaining mobility, balance and cognitive function. The results from the third round of the Delphi process highlighted critical insights into the consensus and discrepancies between health professional experts and hearing-impaired older adults. Notably, there were significant differences in the perceived importance of various training components, particularly those related to motor functions, hearing capabilities and DT training.

### Training components and characteristics

Consensus across both participant groups underscored the inclusion of traditional falls prevention exercises (including walking, strength and balance exercises), confirming their evidence-based role in physical health [30, 31]. Moreover, DT training was rated to be highly relevant, which helps to increase cognitive and motor performance [17, 32, 33].

Distinct preferences became apparent in the assessment of specific exercises. Health professionals frequently rated complex exercises like “Combined strength and balance exercises for fall prevention” and “Exercises with closed eyes” higher than the hearing-impaired participants. This disparity suggests a professional inclination towards comprehensive, sensory-challenging protocols that may not resonate as strongly with hearing-impaired individuals, who might view these exercises as overly demanding or inaccessible without additional support [34, 35].

For the hearing-related aspects, items such as “Listening exercises with background noise”, “Exercises with and without hearing aids”, and “Exercises for distinguishing pitch” received higher ratings from health professionals, reflecting a recognition of the importance of practicing in varied auditory environments to enhance real-world functional hearing [36, 37]. However, hearing-impaired participants often rated these tasks lower, possibly indicating perceived difficulties or lesser relevance, highlighting a gap between expert recommendations and user preferences [38].

For the DT component, the items “Listening tasks during balance exercises” and “Everyday exercises” (e.g., walking and using the phone) also exhibited significant differences. Health professionals rated these dual-task exercises higher, emphasizing the integration of cognitive and physical tasks more than the hearing-impaired participants, who might prioritise immediate auditory comfort over potential cognitive benefits [39]. On the other hand, hearing-impaired participants may have been less inclined to view these exercises as necessary, especially if they perceive balance and cognitive training to be secondary to their hearing needs [38].

The exclusion of “Strength exercises (e.g., squats)” from the program due to low ratings underscores a consensus that these may not be essential for the targeted training goals of hearing-impaired older adults. Similarly, the omission of specified frequent training sessions reflects practical concerns about the feasibility of intensive regimens for this group, highlighting the need for adaptable training schedules that accommodate individual capabilities and lifestyle needs [40, 41]. However, the results indicate, that more knowledge on exercise physiology might benefit future applications. Frequent training sessions stimulate neuroplasticity, enhancing both cognitive and motor functions, which is crucial for older adults facing sensory deficits. The intensity of the exercises should match the individual’s abilities, gradually increasing to ensure engagement without overwhelming participants. For those with hearing impairments, exercises should challenge their cognitive abilities, especially since auditory deficits may require additional focus on visual or tactile cues [42]. The duration of sessions and total training time must be balanced to avoid fatigue while still providing adequate stimulation for cognitive and physical benefits. The types of exercises should be varied, incorporating cognitive and motor tasks tailored to each individual’s needs, particularly considering hearing loss. Moreover, individualisation ensures that training programs are personalised to the participant’s specific cognitive and physical abilities, increasing engagement and effectiveness [43]. Moreover, personalised approaches might reduce frustration and improve long-term adherence to training. These aspects might also align with feedback and training instruction.

### Training Instruction, feedback and monitoring

Feedback preferences within the study revealed a significant trend: hearing-impaired participants particularly valued the option for “Feedback available at any time”. This preference underscores the importance of adaptable feedback mechanisms that can be accessed on-demand, aligning with the fluctuating needs and capacities of hearing-impaired older adults. Such flexibility not only enhances user autonomy but also accommodates the variable nature of hearing impairments, which may affect a person’s ability to engage with the training program consistently [44]. Conversely, the relatively low interest in “Presentation of performance curves” and “Feedback after each exercise” suggests a nuanced approach to feedback is necessary, especially as the variability in the response behavior was rather high. These findings indicate a preference for less frequent feedback that does not overwhelm or disrupt the training experience. This could be particularly relevant for hearing-impaired users who might find continuous feedback too intrusive or distracting, especially if it relies on auditory cues that are less accessible to them. Developers might consider implementing customisable feedback options that allow users to select how and when they receive updates about their progress, thus tailoring the experience to individual preferences and needs.

### Requirements and barriers to app use

The study highlighted the critical importance of ease of use and accessibility in App design, with both hearing-impaired participants and health professionals prioritising these aspects. Simple, intuitive App interfaces are essential, particularly for older adults who may not be as technologically savvy or who might face additional challenges due to hearing impairments. This is reflected by the hearing-impaired older adults rating the item “low self-efficacy” as the greatest barrier. Apps for this target group need to enable and empower their users to keep the motivation high. The significant differences in ratings regarding “Poor user-friendliness” and “High complexity of the App operation” suggest that while hearing-impaired users may find certain technological interfaces manageable, these factors still represent significant barriers that can hinder the effective use of mHealth solutions [45, 46]. Developers must focus on reducing the complexity of the App’s operation, ensuring that features are straightforward and that help and support resources are readily available. Simplifying navigation and minimising the need for frequent adjustments or settings changes can make Apps more accessible and less intimidating for users with sensory impairments. Furthermore, ensuring that Apps are compatible with assistive technologies, such as screen readers or enhanced visual aids, can help bridge the gap between functional design and user needs.

### Data protection and safety

Data protection and safety emerged as a unanimous concern among all study participants, underscoring the universal importance of securing sensitive personal information and ensuring safe use of the App and personal data. This emphasis is crucial not only for building trust but also for complying with legal standards concerning data security and privacy. Participants indicated a strong preference for features that ensure the confidentiality and integrity of data, such as secure login processes, encrypted data storage, and clear privacy policies that outline how data is collected, used, and shared. Additionally, safety features within the App, such as emergency contacts, alert systems for abnormal health readings, and easy access to support in case of difficulties during exercises, are vital. These features can provide users with the confidence to engage fully with the training program, knowing that safeguards are in place to protect them during use. This aspect is particularly critical for older adults who may be at increased risk of falls or other injuries during physical activities. Developers should incorporate robust safety protocols and consider integrating features that monitor user engagement and provide alerts or guidance when potentially risky situations are detected.

### Limitations

This Delphi study has several limitations that could influence its outcomes and generalisability. Firstly, the diversity and size of the panel may limit the findings, comprising a specific group of health professionals and hearing-impaired older adults, therefore may not represent wider perspectives. Hearing loss and balance issues were self-reported, and their severity was not objectively assessed. Additionally, the response rates, particularly the low completion rate for the first round, might have skewed the consensus, affecting the reliability of the results. This also resulted in the problems to gain answers for the second round leading to a delay for the final round.

Geographical limitations are notable since the participant pool was international with regards to the experts but located in Germany only for affected persons, possibly not reflecting the needs and conditions of broader, diverse populations in lower to middle income countries. It is important to recognize that evidence-based practice often relies on international guidelines and recommendations developed by interdisciplinary and global experts in the field of research or public health. In our study, we compared the recommendations of this international expert group with the needs of a German population of older adults with hearing impairments. If the findings of this study are to be applied to other countries or populations, the specific needs of those target groups should be carefully reconsidered.

Lastly, the study’s reliance on technology for conducting surveys might have excluded those with limited access to digital resources or less familiarity with such tools, further affecting the inclusiveness and breadth of the data collected.

### Study implications

The study fills critical knowledge gaps by providing a better understanding on exercise preferences and needs of older adults with hearing impairments, highlighting the need of tailoring interventions to accommodate both the physical and sensory limitations. The findings provide an empirical basis for developing tailored, user-centered cognitive-motor training programs. Exercise professionals can use this information to refine their training protocols, incorporating preferred activities such as exercises involving music and adjustable intensity into their routines, which have been identified as less demanding and more enjoyable by hearing-impaired participants. Furthermore, technology represents a potential avenue for personalising training experiences. Future research should focus on developing and testing advanced assistive technologies that improve the accessibility and efficacy of these training programs with feasibility trials and randomized controlled trials. It is essential for developers to implement robust safety protocols and to include features that alert users to potential risks during exercises. Conducting clinical trials to explore the long-term impacts of these tailored programs on health outcomes, self-efficacy and quality of life are needed to evaluate their effectiveness.

## Conclusions

This Delphi study identified key features and preferences for a cognitive-motor training system designed for older adults with hearing impairments. The training should integrate (1) Flexibility exercises, (2) moment tasks combined with visual or coordination tasks or (3) combinations incorporating strength, endurance, flexibility and balance. Moreover, the findings emphasise the need for personalised, user-centred design that integrates both professional recommendations and the lived experiences of the target population. Programs should be adaptable, easy to use, and respectful of sensory limitations, offering functional benefit, feedback and enjoyable engagement. By incorporating these principles, mHealth interventions can support the mobility, independence, and quality of life of hearing-impaired older adults.

## Supplementary Information

Supplementary material 1.

## Data Availability

No datasets were generated or analysed during the current study.

## Abbreviations

UCD: User-centered design
DT: Dual-task
