# Supplementary material for: Requirements of health professionals and affected persons for an App-based dual-task training for hearing impaired older adults - a Delphi survey
Source: Eur Rev Aging Phys Act. 2025 Oct 23;22:18. doi: 10.1186/s11556-025-00386-7 (PMC12548227; doi:10.1186/s11556-025-00386-7)
Supplement: Supplementary file 1 — Supplementary material 1. [file 11556_2025_386_MOESM1_ESM.pdf]

		affected persons				experts				total				
question	Item	Mean	Number valid responses	responses < 7	percentage responses > 6	Mean	Number valid responses	responses < 7	percentage responses > 6	Mean	SD	Number valid responses	responses < 7	percentage responses > 6
physical training or exercises	Everyday training tasks (e.g., getting up from a chair), functional training	**8.71**	14	2	85.71	**9.25**	12	0	100.00	**8.96**	**1.72**	26	2	**92.31**
	Endurance exercises (e.g., walking)	**9.29**	14	1	92.86	**8.62**	13	2	84.62	**8.96**	**1.48**	27	3	**88.89**
	Flexibility exercises (e.g., gymnastics; specific exercises for foot flexibility)	**9.29**	14	0	100.00	**6.46**	13	7	46.15	**7.93**	**2.11**	27	7	**74.07**
	Strength exercises (e.g., squats)	**7.57**	14	5	64.29	**8.00**	13	3	76.92	**7.78**	**1.81**	27	8	**70.37**
	Balance exercises (e.g., longer one-legged stance or shifting positions from right to left leg)	**9.23**	13	0	100.00	**9.31**	13	0	100.00	**9.27**	**0.81**	26	0	**100.00**
	Movement combined with visual tasks (e.g., walking and observing something)	**8.46**	13	2	84.62	**8.00**	13	2	84.62	**8.23**	**1.72**	26	4	**84.62**
	Movement combined with coordination tasks (e.g., combining walking with arm movements)	**8.71**	14	1	92.86	**8.85**	13	1	92.31	**8.78**	**1.47**	27	2	**92.59**
	Exergames (computer-based games that promote movement, especially balance and strength)	**6.85**	13	5	61.54	**7.83**	12	2	83.33	**7.32**	**2.43**	25	7	**72.00**
	Exercises with closed eyes	**8.14**	14	4	71.43	**7.85**	13	2	84.62	**8.00**	**2.09**	27	6	**77.78**
	Reaction tasks to movements at the periphery of the field of view	**7.86**	14	3	78.57	**6.92**	13	7	46.15	**7.41**	**1.95**	27	10	**62.96**
	Combined strength and balance exercises for fall prevention	**9.00**	14	0	100.00	**9.15**	13	0	100.00	**9.07**	**1.02**	27	0	**100.00**
	Combined training exercises with strength, endurance, flexibility, and balance	**9.21**	14	0	100.00	**9.31**	13	0	100.00	**9.26**	**0.80**	27	0	**100.00**
hearing training or exercises	Everyday exercises (e.g., understanding content from a phone call)	**7.85**	13	2	84.62	**9.11**	9	1	88.89	**8.36**	**2.12**	22	3	**86.36**
	Exercises with music	**7.15**	13	4	69.23	**6.67**	9	4	55.56	**6.95**	**2.36**	22	8	**63.64**
	Listening to audiobooks	**6.69**	13	5	61.54	**5.38**	8	6	25.00	**6.19**	**2.87**	21	11	**47.62**
	Combination of multiple concurrent listening tasks (e.g., tracking different sound sequences)	**6.46**	13	6	53.85	**8.67**	9	0	100.00	**7.36**	**2.25**	22	6	**72.73**
	Exercises with and without hearing aids	**7.00**	12	3	75.00	**7.89**	9	2	77.78	**7.38**	**2.73**	21	5	**76.19**
	Reaction exercises (e.g., responding quickly when a specific sound is heard)	**7.54**	13	4	69.23	**7.88**	8	3	62.50	**7.67**	**2.38**	21	7	**66.67**
	Exercises for distinguishing pitch	**7.31**	13	4	69.23	**8.83**	6	0	100.00	**7.79**	**2.21**	19	4	**78.95**
	Exercises for recognizing sound patterns or noises	**8.46**	13	2	84.62	**9.33**	9	1	88.89	**8.82**	**1.43**	22	3	**86.36**
	Exercises for recognizing consonants	**7.85**	13	3	76.92	**9.00**	6	1	83.33	**8.21**	**2.28**	19	4	**78.95**
	Exercises for distinguishing multiple voices	**8.69**	13	2	84.62	**9.43**	7	0	100.00	**8.95**	**1.50**	20	2	**90.00**
	Listening exercises with background noise	**8.85**	13	2	84.62	**9.11**	9	0	100.00	**8.95**	**1.49**	22	2	**90.91**
	Exercises that involve both brain hemispheres (e.g., recognizing direction of a sound)	**8.75**	12	2	83.33	**9.63**	8	0	100.00	**9.10**	**2.34**	20	2	**90.00**
dual-task training	Navigating complex environments (e.g., crossing a street)	**8.83**	12	1	91.67	**7.54**	13	4	69.23	**8.16**	**2.13**	25	5	**80.00**
	Having conversations while walking	**8.69**	13	1	92.31	**7.83**	12	3	75.00	**8.28**	**1.80**	25	4	**84.00**
	Listening tasks during balance exercises (static, e.g., standing on one leg for an extended period, and dynamic, e.g., switching from right to left leg)	**7.92**	13	3	76.92	**7.92**	13	3	76.92	**7.92**	**2.23**	26	6	**76.92**
	Reacting to visual stimuli during balance exercises (static and dynamic)	**7.77**	13	3	76.92	**6.69**	13	6	53.85	**7.23**	**2.28**	26	9	**65.38**
	Math tasks during balance exercises (static and dynamic)	**7.69**	13	4	69.23	**5.92**	13	6	53.85	**6.81**	**2.42**	26	10	**61.54**
	Exercises in combination with health information	**6.50**	12	7	41.67	**4.91**	11	7	36.36	**5.74**	**2.54**	23	14	**39.13**
	Coordination tasks during balance exercises (static and dynamic)	**7.46**	13	4	69.23	**6.62**	13	5	61.54	**7.04**	**2.61**	26	9	**65.38**
	Performing exercises with music or background noise	**8.08**	13	2	84.62	**6.09**	11	5	54.55	**7.17**	**2.56**	24	7	**70.83**
	Exergames: computer-based games that promote movement (e.g., combining balance and hearing abilities)	**7.25**	12	3	75.00	**8.00**	13	3	76.92	**7.64**	**2.30**	25	6	**76.00**
	Everyday exercises (e.g., walking and using the phone)	**7.92**	13	3	76.92	**7.23**	13	4	69.23	**7.58**	**2.44**	26	7	**73.08**
	Repeating words while walking or doing balance tasks	**7.62**	13	4	69.23	**7.80**	10	3	70.00	**7.70**	**2.23**	23	7	**69.57**
	Exercises in combination with self-efficacy training (gradually building confidence in one's abilities)	**8.36**	11	2	81.82	**7.83**	12	3	75.00	**8.09**	**2.43**	23	5	**78.26**
training frequency	Daily	**7.55**	11	2	81.82	**8.33**	12	2	83.33	**7.96**	**2.42**	23	4	**82.61**
	Daily for 20-40 minutes	**6.80**	10	2	80	**8.00**	12	4	66.67	**7.45**	**2.73**	22	6	**72.73**
	Daily for 20 minutes but with high concentration	**6.30**	10	4	60	**6.75**	12	6	50.00	**6.55**	**2.57**	22	10	**54.55**
	4-5 times per week	**6.92**	12	5	58.33	**8.67**	12	1	91.67	**7.79**	**2.14**	24	6	**75.00**
	2-3 times per week	**5.92**	12	7	41.67	**6.42**	12	6	50.00	**6.17**	**3.32**	24	13	**45.83**
	Once a week	**2.73**	11	10	9.09	**3.64**	11	9	18.18	**3.18**	**2.90**	22	19	**13.64**

[illegible]	Following the simple WHO criteria: 150 minutes of training and at least five times per week 30 minutes of exercise	**7.23**	13	5	61.54	**7.91**	11	3	72.73	**7.54**	**2.83**	24	8	**66.67**
	Following the comprehensive WHO criteria for older adults: at least five times per week 30 minutes of exercise plus 150-300 minutes per week of endurance training plus 120 minutes of balance training plus two strength training sessions per week	**6.50**	12	5	58.33	**8.18**	11	2	81.82	**7.30**	**2.59**	23	7	**69.57**
**training duration**	30-60 minutes	**6.33**	12	5	58.33	**5.00**	10	7	30.00	**5.73**	**3.26**	22	12	**45.45**
	60-90 minutes	**5.45**	11	5	54.55	**5.40**	10	6	40.00	**5.43**	**3.03**	21	11	**47.62**
	90-150 minutes	**6.42**	12	6	50.00	**7.17**	12	4	66.67	**6.79**	**2.87**	24	10	**58.33**
	At least 150 minutes	**6.00**	11	5	54.55	**7.70**	10	3	70.00	**6.81**	**3.19**	21	8	**61.90**
	Training cycle of at least 20 weeks	**8.91**	11	1	90.91	**8.70**	10	2	80.00	**8.81**	**1.87**	21	3	**85.71**
**training control measures**	Regular tests to assess individual physical fitness and hearing ability	**8.50**	12	2	83.33	**8.83**	12	2	83.33	**8.67**	**1.75**	24	4	**83.33**
	Adjustment of training volume (e.g., duration of training sessions) based on individual physical and cognitive performance	**8.92**	13	2	84.62	**9.00**	12	0	100.00	**8.96**	**1.43**	25	2	**92.00**
	Adjustment of training intensity (e.g., complexity of tasks) based on individual physical performance and hearing ability	**8.23**	13	3	76.92	**9.42**	12	0	100.00	**8.80**	**2.06**	25	3	**88.00**
	Adjustment of training volume (e.g., duration of training sessions) based on World Health Organization (WHO) recommendations	**6.42**	12	6	50.00	**8.17**	12	2	83.33	**7.29**	**2.59**	24	8	**66.67**
	Adjustment of training volume (e.g., duration of training sessions) based on recommendations for fall prevention	**7.69**	13	3	76.92	**8.75**	12	0	100.00	**8.20**	**2.06**	25	3	**88.00**
	Increase in training volume and duration based on individual improvements	**8.54**	13	3	76.92	**8.67**	12	2	83.33	**8.60**	**2.23**	25	5	**80.00**
	Increase in training difficulty based on individual improvements	**8.62**	13	2	84.62	**9.83**	12	0	100.00	**9.20**	**1.41**	25	2	**92.00**
	Recording of training participation	**7.36**	11	3	72.73	**8.45**	11	2	81.82	**7.91**	**2.52**	22	5	**77.27**
**Instruction presentation**	Text (written)	**7.09**	11	4	63.64	**6.45**	11	5	54.55	**6.77**	**2.75**	22	9	**59.09**
	Text (spoken)	**6.50**	12	6	50.00	**6.27**	11	5	54.55	**6.39**	**2.86**	23	11	**52.17**
	Images	**7.82**	11	3	72.73	**8.00**	11	2	81.82	**7.91**	**2.19**	22	5	**77.27**
	Combination of images and text	**7.91**	11	3	72.73	**9.17**	12	1	91.67	**8.57**	**2.24**	23	4	**82.61**
	Short videos	**8.75**	12	2	83.33	**8.45**	11	1	90.91	**8.61**	**2.12**	23	3	**86.96**
	Combination of short videos and explanatory text	**9.00**	12	1	91.67	**9.58**	12	0	100.00	**9.29**	**1.24**	24	1	**95.83**
**Feedback content**	Correction during the execution of exercises	**8.50**	12	2	83.33	**8.67**	9	1	88.89	**8.57**	**1.76**	21	3	**85.71**
	Presentation of performance curves	**7.45**	11	5	54.55	**8.89**	9	0	100.00	**8.10**	**2.53**	20	5	**75.00**
	Feedback on current exercise progress (e.g., number of exercises completed)	**8.58**	12	2	83.33	**9.22**	9	0	100.00	**8.86**	**1.42**	21	2	**90.48**
	Feedback on current performance	**8.50**	12	2	83.33	**8.89**	9	0	100.00	**8.67**	**1.43**	21	2	**90.48**
	Feedback on current performance compared to the goal	**8.17**	12	3	75.00	**8.89**	9	0	100.00	**8.48**	**1.92**	21	3	**85.71**
	Feedback on the difficulty level of the exercise	**8.08**	12	3	75.00	**8.13**	8	1	87.50	**8.10**	**2.37**	20	4	**80.00**
	No feedback	**2.44**	9	8	11.11	**1.25**	8	8	0.00	**1.88**	**2.05**	17	16	**5.88**
**Feedback presentation**	Timing: Weekly	**7.55**	11	3	72.73	**6.88**	8	2	75.00	**7.26**	**2.45**	19	5	**73.68**
	After each session	**8.00**	8	2	75.00	**7.78**	9	2	77.78	**7.88**	**2.72**	17	4	**76.47**
	After each exercise	**7.44**	9	2	77.78	**7.10**	10	3	70.00	**7.26**	**2.86**	19	5	**73.68**
	Available at any time	**8.67**	9	2	77.78	**9.44**	9	0	100.00	**9.06**	**1.39**	18	2	**88.89**
	Presentation format: Auditory (e.g., via a voice function in the app)	**6.67**	9	5	44.44	**5.90**	10	5	50.00	**6.26**	**3.14**	19	10	**47.37**
	Visual (e.g., pop up of points gained)	**9.09**	11	1	90.91	**9.50**	10	0	100.00	**9.29**	**1.24**	21	1	**95.24**
**App functions**	Options for performing exercises together with others	**4.30**	10	7	30.00	**7.00**	10	4	60.00	**5.65**	**3.00**	20	11	**45.00**
	Buttons for repeating instructions	**8.36**	11	1	90.91	**9.70**	10	0	100.00	**9.00**	**1.98**	21	1	**95.24**
	Option to select the format (verbal, video, or written) for exercise instructions	**9.45**	11	0	100.00	**8.90**	10	1	90.00	**9.19**	**1.05**	21	1	**95.24**
	Option to select the format (verbal, video, or written) for exercise feedback	**8.55**	11	1	90.91	**8.90**	10	1	90.00	**8.71**	**2.03**	21	2	**90.48**
	Gamification elements (e.g., high scores, rewards for participation and achievements, etc.)	**7.33**	9	3	66.67	**9.00**	10	0	100.00	**8.21**	**2.28**	19	3	**84.21**
	Specific elements to enhance motivation (e.g., positive messages)	**7.67**	9	2	77.78	**7.80**	10	2	80.00	**7.74**	**2.47**	19	4	**78.95**
	Competitions with other users	**9.11**	9	0	100.00	**7.00**	10	3	70.00	**8.00**	**2.41**	19	3	**84.21**
	Short interesting or amusing facts about movement, balance, hearing, etc.	**4.33**	9	7	22.22	**6.60**	10	4	60.00	**5.53**	**2.76**	19	11	**42.11**
	Music	**7.10**	10	4	60.00	**7.10**	10	3	70.00	**7.10**	**2.51**	20	7	**65.00**
	Opportunities for networking with medical care (e.g., personal counseling)	**8.00**	9	1	88.89	**6.60**	10	4	60.00	**7.26**	**2.36**	19	5	**73.68**
	Feedback to clinical personnel on the number of completed trainings	**6.38**	8	4	50.00	**7.10**	10	3	70.00	**6.78**	**2.68**	18	7	**61.11**
	Networking with other users	**4.11**	9	7	22.22	**6.50**	10	4	60.00	**5.37**	**3.15**	19	11	**42.11**
	Reminder function	**7.60**	10	3	70.00	**8.50**	10	1	90.00	**8.05**	**2.33**	20	4	**80.00**
	Display of current heart rate and calorie expenditure	**7.00**	9	3	66.67	**6.20**	10	5	50.00	**6.58**	**2.50**	19	8	**57.89**

	Information material on hearing impairment and balance difficulties	**7.40**	10	2	80.00	**7.80**	10	1	90.00	**7.60**	**2.08**	20	3	**85.00**
	Information on how training adjustments are made	**8.00**	10	2	80.00	**7.80**	10	3	70.00	**7.90**	**2.19**	20	5	**75.00**
	Information on techniques for behavior change	**8.40**	10	2	80.00	**7.50**	10	4	60.00	**7.95**	**1.69**	20	6	**70.00**
	Ease of use	**9.73**	11	0	100.00	**9.90**	10	0	100.00	**9.81**	**0.39**	21	0	**100.00**
	Exercises that are enjoyable	**9.64**	11	0	100.00	**8.90**	10	1	90.00	**9.29**	**1.45**	21	1	**95.24**
	Simple barrier-free design (e.g., large letters and input buttons)	**9.64**	11	0	100.00	**9.80**	10	0	100.00	**9.71**	**0.55**	21	0	**100.00**
	Easily understandable instructions	**9.64**	11	0	100.00	**9.70**	10	0	100.00	**9.67**	**0.64**	21	0	**100.00**
	Simple graphics for monitoring progress	**9.45**	11	0	100.00	**9.10**	10	0	100.00	**9.29**	**0.82**	21	0	**100.00**
	Support for learning how to use the app upon initial use	**9.45**	11	0	100.00	**8.90**	10	1	90.00	**9.19**	**1.18**	21	1	**95.24**
	User manual	**9.36**	11	0	100.00	**7.90**	10	1	90.00	**8.67**	**2.03**	21	1	**95.24**
**App usability**	Option to receive technical support	**9.64**	11	0	100.00	**8.80**	10	1	90.00	**9.24**	**1.19**	21	1	**95.24**
	Simple tests to assess physical fitness	**9.73**	11	0	100.00	**9.10**	10	0	100.00	**9.43**	**0.79**	21	0	**100.00**
	Simple tests to assess hearing ability	**7.67**	9	3	66.67	**9.50**	10	0	100.00	**8.63**	**1.56**	19	3	**84.21**
	Instructions for creating a safe training environment	**9.63**	8	0	100.00	**8.33**	9	2	77.78	**8.94**	**1.55**	17	2	**88.24**
	Options for integrating personal hearing aids	**8.50**	8	1	87.50	**9.00**	8	0	100.00	**8.75**	**1.09**	16	1	**93.75**
	Movement tracking	**9.82**	11	0	100.00	**7.40**	10	2	80.00	**8.67**	**1.58**	21	2	**90.48**
	Compatibility with existing devices	**9.82**	11	0	100.00	**8.56**	9	2	77.78	**9.25**	**1.51**	20	2	**90.00**
	Option for training without additional functions	**8.50**	10	1	90.00	**7.67**	9	1	88.89	**8.11**	**3.03**	19	2	**89.47**
	Number of steps per day	**8.18**	11	2	81.82	**7.60**	10	3	70.00	**7.90**	**2.41**	21	5	**76.19**
	Risk of falls	**8.64**	11	1	90.91	**8.70**	10	1	90.00	**8.67**	**1.43**	21	2	**90.48**
	Postural stability/balance	**9.00**	11	1	90.91	**9.60**	10	0	100.00	**9.29**	**1.12**	21	1	**95.24**
**motor function to monitor**														
	Combination of strength, balance, and gait (e.g., SPPB or TUG)	**9.27**	11	0	100.00	**9.20**	10	0	100.00	**9.24**	**0.68**	21	0	**100.00**
	Gait analysis (e.g., speed, posture, safety)	**9.45**	11	0	100.00	**8.30**	10	1	90.00	**8.90**	**1.23**	21	1	**95.24**
	Leg mobility	**9.18**	11	1	90.91	**6.40**	10	5	50.00	**7.86**	**2.38**	21	6	**71.43**
	Sound localization	**8.40**	10	1	90.00	**9.33**	6	0	100.00	**8.75**	**2.19**	16	1	**93.75**
	Speech discrimination	**8.73**	11	1	90.91	**9.40**	5	0	100.00	**8.94**	**2.19**	16	1	**93.75**
	Sensitivity to tone discrimination	**8.50**	10	1	90.00	**8.60**	5	0	100.00	**8.53**	**2.25**	15	1	**93.33**
**hearing function to monitor**	Word recognition in background noise	**9.78**	9	0	100.00	**9.20**	5	0	100.00	**9.57**	**0.62**	14	0	**100.00**
	Auditory memory	**9.11**	9	0	100.00	**8.20**	5	0	100.00	**8.79**	**1.08**	14	0	**100.00**
	Hearing everyday sounds, hearing threshold (e.g., PTA)	**9.44**	9	0	100.00	**8.83**	6	0	100.00	**9.20**	**0.83**	15	0	**100.00**
	Hearing everyday sounds despite various background noises	**9.55**	11	0	100.00	**9.17**	6	0	100.00	**9.41**	**0.97**	17	0	**100.00**
	Recognition of the content of spoken words	**9.40**	10	0	100.00	**8.60**	5	0	100.00	**9.13**	**0.96**	15	0	**100.00**
	Reaction speed to signals	**9.45**	11	0	100.00	**8.83**	6	1	83.33	**9.24**	**1.21**	17	1	**94.12**
	Low self-efficacy	**6.83**	6	2	66.67	**8.30**	10	1	90.00	**7.75**	**2.25**	16	3	**81.25**
	Low willingness to use smartphones	**6.90**	10	4	60.00	**8.60**	10	0	100.00	**7.75**	**2.68**	20	4	**80.00**
	Poor user-friendliness	**8.50**	8	2	75.00	**9.20**	10	0	100.00	**8.89**	**2.54**	18	2	**88.89**
	Incompatibility of the app with older smartphone models	**7.33**	9	4	55.56	**8.50**	10	1	90.00	**7.95**	**2.44**	19	5	**73.68**
	Low motivation (e.g., to train)	**7.13**	8	4	50.00	**7.90**	10	3	70.00	**7.56**	**1.89**	18	7	**61.11**
	No person to monitor safety	**6.88**	8	3	62.50	**6.10**	10	4	60.00	**6.44**	**2.52**	18	7	**61.11**
	Lack of clear or absent rationale for recommendations	**8.75**	8	1	87.50	**7.30**	10	2	80.00	**7.94**	**1.84**	18	3	**83.33**
	Time and opportunity	**8.63**	8	1	87.50	**7.67**	9	3	66.67	**8.12**	**2.11**	17	4	**76.47**
**barriers of an App**	Too high complexity of the app and its operation	**8.88**	8	1	87.50	**9.00**	10	1	90.00	**8.94**	**1.90**	18	2	**88.89**
	Visual impairment	**7.71**	7	2	71.43	**8.86**	7	1	85.71	**8.29**	**3.47**	14	3	**78.57**
	Lack of technology/equipment	**8.22**	9	3	66.67	**8.40**	10	2	80.00	**8.32**	**2.13**	19	5	**73.68**
	No sense of belonging to the target group	**6.75**	8	4	50.00	**7.50**	10	4	60.00	**7.17**	**2.43**	18	8	**55.56**
	Missing or delayed feedback on personal progress	**7.75**	8	3	62.50	**7.30**	10	4	60.00	**7.50**	**2.03**	18	7	**61.11**
	Boredom	**6.43**	7	4	42.86	**7.38**	8	3	62.50	**6.93**	**2.43**	15	7	**53.33**
	Feeling overwhelmed/Frustration	**8.25**	8	2	75.00	**7.90**	10	3	70.00	**8.06**	**2.50**	18	5	**72.22**
	Lack of data privacy	**6.88**	8	4	50.00	**5.60**	10	5	50.00	**6.17**	**3.06**	18	9	**50.00**
	Safety instructions for use	**8.82**	11	1	90.91	**7.90**	10	1	90.00	**8.38**	**2.42**	21	2	**90.48**
**safety aspects (question 13 & 15)**	Safety instructions during training, warnings	**8.73**	11	1	90.91	**8.10**	10	2	80.00	**8.43**	**2.59**	21	3	**85.71**
	Legal data protection	**8.09**	11	2	81.82	**7.20**	10	4	60.00	**7.67**	**2.85**	21	6	**71.43**
	Information about the collection and use of personal data	**8.45**	11	2	81.82	**7.40**	10	3	70.00	**7.95**	**2.85**	21	5	**76.19**
	Training participation/duration	**6.40**	10	4	60.00	**8.80**	10	0	100.00	**7.60**	**2.31**	20	4	**80.00**
	Physical fitness	**7.70**	10	3	70.00	**8.30**	10	0	100.00	**8.00**	**1.45**	20	3	**85.00**
**interface to professionals**	User satisfaction feedback	**8.40**	10	2	80.00	**7.50**	10	2	80.00	**7.95**	**1.86**	20	4	**80.00**
	Training results/success	**7.33**	9	3	66.67	**9.50**	10	0	100.00	**8.47**	**1.79**	19	3	**84.21**
	Thresholds for clinically relevant changes	**8.88**	8	0	100.00	**9.30**	10	0	100.00	**9.11**	**0.94**	18	0	**100.00**
